# Stiripentol directly attenuates immunosuppression of tumor-infiltrating myeloid cells to potentiate anti-PD-1 efficacy

**DOI:** 10.1186/s12885-026-15793-x

**Published:** 2026-02-28

**Authors:** Zhiqi Xie, Kexin Wang, Jiayi Liu, Ziyi Hong, Yuanzhen Jin, Linying Wang, Masashi Tachibana, Jian Liu, Meihua Lin, Zeren Shen, Jinjin Shao

**Affiliations:** 1 Wuyi First People’s Hospital, Affiliated Hospital, School of Medicine, Hangzhou City University, Hangzhou, 310015 China; 2 School of Medicine, Hangzhou City University, Hangzhou, 310015 China; 3 Key Laboratory of Drug Safety Evaluation and Research of Zhejiang Province, Center of Safety Evaluation and Research, Hangzhou Medical College, Hangzhou, 310053 China; 4 Department of Plastic Surgery, The First Affiliated Hospital, Zhejiang University School of Medicine, Hangzhou, 310003 China; 5 Laboratory for Context-Dependent Cell Immunology, Department of Biomedical Sciences, College of Life Sciences, Ritsumeikan University, 1-1-1 Nojihigashi, Kusatsu, Shiga 525-8577 Japan; 6 Department of Clinical Pharmacy, The First Affiliated Hospital, Zhejiang University School of Medicine, Hangzhou, 310003 China; 7 Zhejiang Key Laboratory of Drug Evaluation and Clinical Research, Hangzhou, 310003 China

**Keywords:** Stiripentol, Myeloid-derived suppressor cells, Tumor-associated macrophages, Immunotherapy, PD-1

## Abstract

**Background:**

Immune checkpoint blockade (ICB) therapy demonstrates significant efficacy across multiple malignancies, yet resistance remains prevalent. Recent studies have repositioned the clinically approved antiepileptic drug stiripentol (STP) as a potent lactate dehydrogenase A inhibitor that effectively disrupts tumor metabolic reprogramming, thereby reinstating tumor control. Despite its promising antineoplastic activity, the direct impact of STP on immune cells and its potential synergy with anti-PD-1 antibodies remain unexplored.

**Methods:**

We used an orthotopic 4T1 breast cancer model to evaluate whether STP remodels the immunosuppressive tumor microenvironment and enhances anti-PD-1 efficacy. Tumor growth was monitored, and immune cell profiles in tumors and blood were analyzed by flow cytometry. Lactate levels and chemokine expression in tumor tissue were also assessed. In vitro, the effects of STP on 4T1 cell proliferation and lactate secretion, bone marrow-derived myeloid-derived suppressor cell (MDSC) differentiation and suppressive function, and macrophage polarization were evaluated.

**Results:**

The combination of STP and anti-PD-1 synergistically suppressed 4T1 tumor growth, reduced the infiltration of immunosuppressive MDSCs and M2-like tumor-associated macrophages, and reactivated CD8⁺ T cells. STP inhibited 4T1 cell proliferation and lactate secretion in a concentration-dependent manner. Furthermore, STP directly reprogrammed MDSCs by inducing MHC II⁺ cell differentiation and attenuating their T cell suppressive capacity via downregulation of immunosuppressive genes. STP also drove M2-to-M1 macrophage repolarization. Importantly, STP exhibited no direct T cell-activating effects.

**Conclusions:**

Our work establishes STP as a potent remodeler of the immunosuppressive tumor microenvironment that synergizes with anti-PD-1 therapy, providing a novel strategy to overcome ICB resistance.

**Supplementary Information:**

The online version contains supplementary material available at 10.1186/s12885-026-15793-x.

## Background

Cancer remains a global health challenge with profound clinical consequences. Central to tumor immune evasion is the PD-1/PD-L1 axis, through which tumor-expressed PD-L1 engages PD-1 on T cells, suppressing their cytolytic function and enabling immune escape [1]. Clinically, immune checkpoint blockade (ICB) therapy demonstrates significant efficacy across diverse malignancies by reactivating antitumor immunity [2, 3]. However, intrinsic or acquired resistance affects a substantial proportion of patients, driven primarily by the immunosuppressive tumor microenvironment (TME) [4, 5]. Within this milieu, myeloid-derived suppressor cells (MDSCs) and tumor-associated macrophages (TAMs) emerge as pivotal orchestrators of immune suppression [6, 7]. As the most abundant immune population in tumors, TAMs secrete Arg1, IL-10, and TGF-β to directly inhibit T and natural killer (NK) cell activity [8, 9]. Similarly, MDSCs deploy arginase, nitric oxide, ROS, and TGF-β to suppress T-cell responses and impair NK cell cytotoxicity [10, 11]. Preclinical and clinical evidence indicates that targeting MDSCs and TAMs enhances tumor control when combined with ICB, suggesting this approach could overcome resistance and improve therapeutic outcomes [12–15].

The lactate-enriched TME further exacerbates immunosuppression by impairing CD8⁺ T-cell function and polarizing myeloid populations toward protumoral phenotypes [16–18]. Notably, the clinically approved antiepileptic drug stiripentol (STP) exhibits pharmacological effects beyond neuroregulation, functioning as a potent lactate dehydrogenase A (LDHA) inhibitor that significantly reduces lactate concentrations in the TME [19–21]. Recent studies demonstrate that STP disrupts tumor metabolic reprogramming and shows synergistic effects when combined with chemotherapy to overcome drug resistance and prolong survival [22–24]. Preclinical evidence confirms that STP-mediated LDHA inhibition reduces lactate levels, thereby improving the metabolic fitness of CD8^+^ T cells [25]. Additionally, STP-induced lactate reduction impacts cancer cell DNA repair and chemoresistance [26]. Nevertheless, the direct effects of STP on immunosuppressive myeloid cells and its potential synergy with PD-1 blockade remain uncharacterized.

Given the promising antineoplastic activity of STP and its unexplored immunomodulatory effects, we hypothesize that this repurposed agent potently remodels the tumor immune landscape and enhances anti-PD-1 efficacy. This study investigates the synergistic antitumor activity and underlying mechanisms of STP combined with anti-PD-1 antibody, with a focus on elucidating the direct impact of STP on immune cell function. Our work aims to provide a novel myeloid-targeted strategy to overcome ICB resistance.

## Methods

### Animals

Female C57BL/6J and BALB/c mice (6–8 weeks old) were obtained from Hangzhou Qizhen Experimental Animal Technology. Mice were acclimatized for 7 days under specific pathogen-free conditions with a 12-h light/dark cycle, *ad libitum* access to food and water, and maintained in barrier facilities.

### Cell culture and conditioned media preparation

4T1 murine breast cancer cells (Meisen CTCC, CTCC-001-0649, CN) were cultured in DMEM (Gibco, US) supplemented with 10% FBS (AusGeneX, AU) and 0.1% penicillin/streptomycin (Gibco) at 37 °C with 5% CO_2_. Mycoplasma-free cells were used for all experiments. For tumor-conditioned media, cells at 80% confluence in 75-cm^2^ flasks were passage into triplicate flasks. After 48 h, supernatants were centrifuged (400 × g, 8 min), sterile-filtered (0.22-µm pore; Millex-GP, US), and stored at − 20℃ for future experiments.

### Tumor models and treatments

4T1 cells (1 × 10^6^ in 100 µL PBS) were orthotopically injected into the right mammary fat pad of BALB/c mice. On day 7 post-inoculation, mice were randomized into treatment cohorts (*n* = 6 per group) receiving: 10 mg/kg anti-PD-1 monoclonal antibody (mAb) (BioXCell, BE0146, US) intraperitoneally every 3 days, with concurrent daily intraperitoneal administration of 150 mg/kg STP (Absin, CN), or vehicle control (days 8–26). The STP dose was selected based on prior studies demonstrating its tolerability and pharmacological activity in murine models [22, 23]. Peripheral blood was collected via the tail vein on days 0, 7, 13, 20, and 27. On day 27, mice were deeply anesthetized with 2% isoflurane and euthanized by cervical dislocation; tumors were immediately excised intact and weighed (Fig. S1A). Excised tissues underwent parallel processing: aliquots were homogenized for lactic acid and chemokine quantification, while others were dissociated into single-cell suspensions for flow-cytometric analysis. Tumor volumes were monitored throughout the study period using calipers and calculated as V = 0.5 × length × (width)².

### Single-cell suspension preparation

Tumors were minced in ice-cold HBSS and digested (37℃, 60 min, 100 rpm) in HBSS with Ca_2_^+^/Mg_2_^+^ containing collagenase IV (1 mg/mL; Worthington, US) and DNase I (0.1 mg/mL; Roche, CH). Digests were filtered through a 70-µm strainer (Absin), centrifuged (300 × g, 5 min, 4℃), and treated with red blood cell lysis buffer (Absin). Cells were washed in FACS buffer (PBS containing 2% FBS) and resuspended at 1 × 10^7^ cells/mL. Peripheral blood mononuclear cells were isolated via two cycles of red blood cell lysis buffer (400 × g, 5 min, 4℃) after collection from the retro-orbital plexus.

### Bone Marrow (BM)-MDSC differentiation

BM cells isolated from C57BL/6J mice were differentiated into MDSCs over 4 days in RPMI-1640 medium (Gibco) with 10% FBS, supplemented with 40 ng/mL recombinant murine GM-CSF (Peprotech, US) and 20% (v/v) 4T1-conditioned medium. Cultures were maintained with or without STP. Differentiated cells were analyzed by flow cytometry, qRT-PCR, and T-cell suppression assays.

### MDSC suppression assay

Splenic CD8⁺ T cells from C57BL/6J mice were isolated using the MojoSort Mouse CD8 T Cell Isolation Kit (BioLegend, US), labeled with eFluor 670 (Thermo Fisher, US), and co-cultured with STP-treated BM-MDSCs at ratios of 1:1, 0.5:1, and 0.25:1 in anti-CD3ε/CD28 coated 96-well plates (1 µg/mL each; BioLegend). After 72 h, T-cell proliferation was assessed via eFluor 670 dilution using flow cytometry.

### T-cell proliferation assay

Isolated CD8⁺ T cells (as described above) were labeled with eFluor 670 and cultured (5 × 10^4^ cells/well) with titrated STP concentrations under CD3ε/CD28 stimulation. After 72 h, cells were treated with GolgiStop (1:1000; BD Biosciences, US) for intracellular cytokine staining. Proliferation (eFluor 670 dilution) and IFN-γ expression were quantified by flow cytometry.

### BM-derived macrophage differentiation

BM cells from C57BL/6J mice were cultured for 7 days in DMEM (Gibco) with 10% FBS, and 20 ng/mL M-CSF (Peprotech) to generate macrophages. M2 polarization was induced overnight with 100 ng/mL IL-4 and 50 ng/mL IL-13 (Novoprotein, CN). Polarized macrophages were treated with STP and 20% (v/v) 4T1-conditioned medium for 24 h, then harvested for flow cytometry and qRT-PCR analysis.

### Quantitative RT-PCR

Total RNA was extracted using the RNeasy Mini Kit (Qiagen). RNA concentration and quality were determined using a NanoOne ultra-micro volume spectrophotometer (Yooning, CN). Reverse transcription was performed using the PrimeScript RT Reagent Kit (Takara, JP). qPCR was conducted using SYBR Premix Ex Taq (Takara) on a CFX96 Touch system (Bio-Rad, USA). Primer sequences are listed in Supplementary Table 1. Relative expression was calculated via 2^(−ΔΔCt) method with *Gapdh* normalization. Melt curve analysis confirmed amplicon specificity.

### Flow cytometry

Single-cell suspensions were blocked with anti-CD16/32 (BioLegend) and stained with the indicated antibodies in FACS buffer. The following antibody panels (from BioLegend or BD Biosciences) were used for different cell types. For BM-MDSCs: APC anti-mouse MHC II, R718 anti-mouse CD11c, BV510 anti-mouse Ly-6C, FITC anti-mouse/human CD11b, PE-Cy7 anti-mouse F4/80, and Pacific Blue anti-mouse Ly-6G; for blood/tumor immune cell suspensions: RB705 anti-mouse CD45, FITC anti-mouse/human CD11b, PE anti-mouse F4/80, APC-Cy7 anti-mouse CD11c, PE-Cy7 anti-mouse CD206, Pacific Blue anti-mouse Ly-6G, APC anti-mouse Ly-6C, APC/Cy7 anti-mouse CD49b, APC anti-mouse CD8a, Brilliant Violet 510 anti-mouse CD4, PE-Cy7 anti-mouse CD69, Brilliant Violet 421 anti-mouse IFN-γ, and AF488 anti-mouse Foxp3; for BM-derived macrophages: APC anti-mouse CD11b, FITC anti-mouse F4/80, Brilliant Violet 421 anti-mouse CD86, and PE-Cy7 anti-mouse CD206. Apoptosis was analyzed using FITC Annexin V and 7-AAD viability staining solution. For intracellular cytokine staining of IFN-γ, cells were incubated with GolgiStop for 4 h at 37℃, followed by fixation and permeabilization using a commercial kit (BD Biosciences). Intracellular staining for Foxp3 and CD206 was also performed using the same fixation/permeabilization approach. All samples were acquired on a BD FACSCanto II flow cytometer and analyzed using FlowJo software (version 10.8.1).

### Cell proliferation assay

4T1 cells (5 × 10^3^/well) were seeded in 96-well plates. After 12 h, cells were treated with STP for 48 h. Cell Counting Kit-8 (CCK-8) reagent (20% v/v; Uelandy, CN) was added for 1 h at 37℃. Absorbance was measured at 450 nm (reference: 600 nm) using a TruReader-E20 full-spectrum microplate reader (Yooning, CN).

### Lactate quantification

For tissue samples, tumors were homogenized in ice-cold lysis buffer, centrifuged (12,000 × g, 10 min, 4℃), and supernatants analyzed using an L-Lactic Acid Kit (Beyotime, CN) via standard curve. For cell culture samples, supernatants from 4T1 cells treated with STP for 48 h were analyzed identically.

### Statistical analysis

Data are presented as the mean ± standard error of the mean. Statistical significance was determined using unpaired two-tailed Student’s t-tests or one-/two-way ANOVA in GraphPad Prism. Significance levels are denoted as follows: ns, not significant; *p < 0.05, **p < 0.01, ***p < 0.001, ****p < 0.0001.

## Results

### STP synergizes with anti-PD-1 therapy to enhance antitumor efficacy and remodel the immunosuppressive TME

**Fig. 1:**
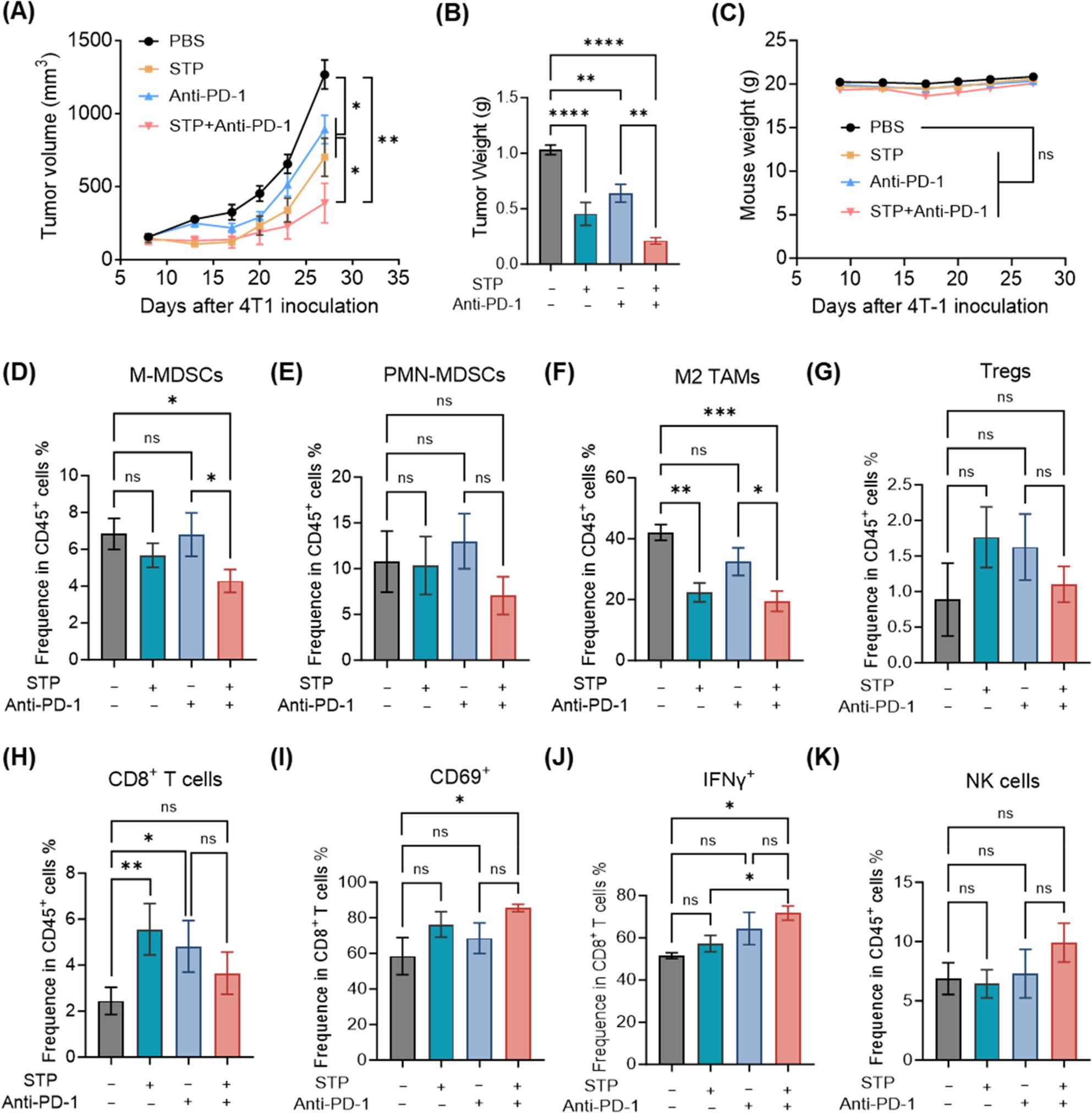
STP enhances anti-tumor efficacy of anti-PD-1 antibody and ameliorates the immunosuppressive tumor microenvironment. **A** Tumor growth curves in 4T1 models treated with STP and anti-PD-1 (*n* = 6). **B** Final tumor weights across treatment groups. **C** Body weight curves of 4T1 tumor-bearing mice. **D**–**K** Flow cytometry analysis of tumor-infiltrating immune cells: M-MDSC (CD45^+^ CD11b^+^ Ly6G^-^ Ly6C^+^), PMN-MDSC (CD45^+^ CD11b^+^ Ly6G^+^ Ly6C^int^), M2-like TAMs (CD45^+^ CD11b^+^ CD11c^-^ F4/80^+^ CD206^+^), Tregs (CD45^+^ CD8^-^ CD4^+^ FoxP3^+^), NK cells (CD45^+^ CD8^-^ CD49b^+^), CD8^+^ T cells (CD45^+^ CD49b^-^ CD8^+^) among CD45^+^ cells, and CD69^+^ cells, IFN-γ^+^ cells among CD8^+^ T cells

We evaluated the antitumor activity of STP combined with anti-PD-1 antibody in an orthotopic 4T1 breast cancer model. While monotherapies modestly inhibited tumor progression, the combination treatment significantly enhanced antitumor efficacy compared to either single agent, markedly reducing tumor growth (Fig. 1A, B). No animal deaths or significant body weight changes were observed in any group (Fig. 1C). Profound remodeling of the tumor immune landscape was observed in the combination group (Fig. S1B): the frequencies of immunosuppressive monocytic MDSCs (M-MDSCs) and M2-polarized TAMs (CD206⁺) were significantly decreased compared with the PBS or anti-PD-1 monotherapy groups, whereas the proportions of polymorphonuclear MDSCs (PMN-MDSCs) and regulatory T cells (Tregs) remained unchanged (Fig. 1D–G). Concomitant with this attenuated myeloid suppression, an increasing trend in the infiltration of CD8⁺ T cells and NK cells was also observed (Fig. 1H, K). Furthermore, CD8⁺ T cells exhibited significantly increased activation (CD69⁺) and effector function (IFN-γ⁺) (Fig. 1I, J). These findings indicate that STP reprograms the immunosuppressive TME, thereby potentiating the efficacy of anti-PD-1 therapy.

**Fig. 2:**
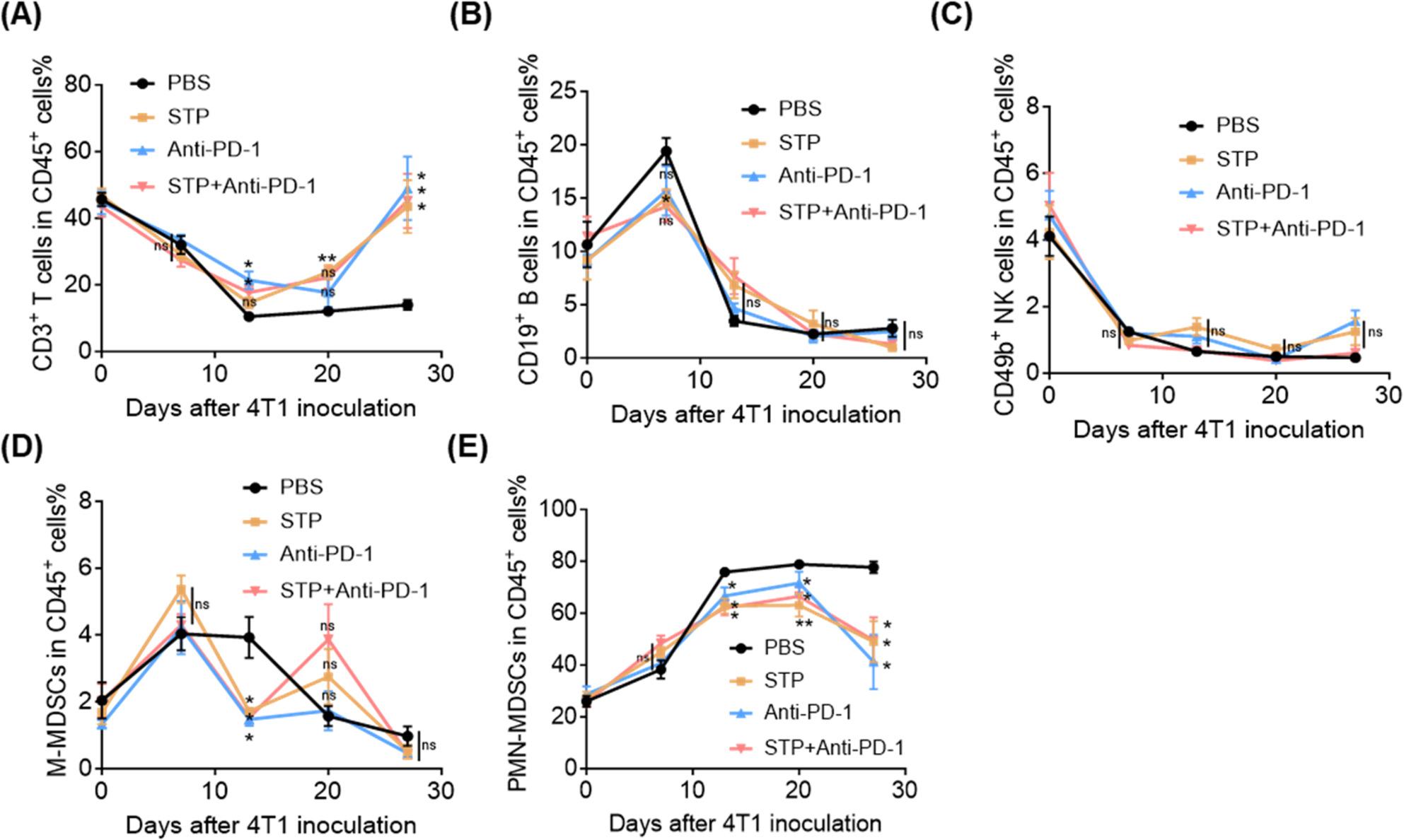
STP combined with anti-PD-1 antibody reduces myeloid cell proportions and increases T cell proportions in peripheral blood. Blood was collected from the tail vein of 4T1 tumor-bearing mice at days 0, 7, 13, 20, and 27 post-tumor inoculation. Flow cytometry was performed to analyze proportions among CD45^+^ cells of (**A**) T cells (CD45^+^ CD3^+^), (**B**) B cells (CD45^+^ CD19^+^), (**C**) NK cells (CD45^+^ CD49b^+^), (**D**) M-MDSC (CD45^+^ CD11b^+^ Ly6G^-^ Ly6C^+^), (**E**) PMN-MDSC (CD45^+^ CD11b^+^ Ly6G^+^ Ly6C^int^)

Longitudinal blood monitoring revealed progressive tumor-induced dysregulation: early expansion of myeloid cells (particularly PMN-MDSCs) and a concomitant decline in T cell populations were counteracted by both monotherapies and the combination treatment (Fig. 2A-E, S1C). This systemic immunomodulatory effect suggests a reversal of tumor-driven hematopoiesis.

**Fig. 3:**
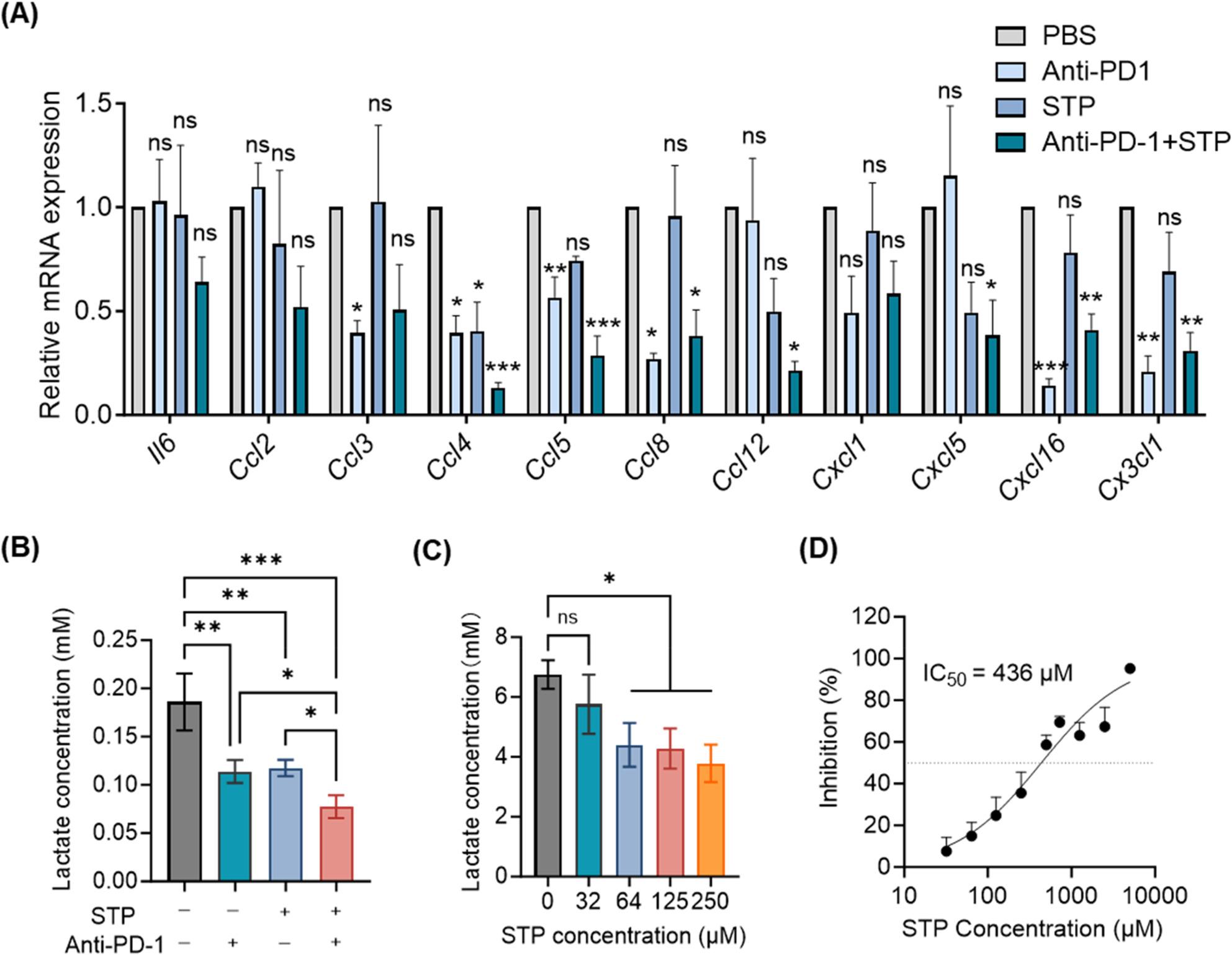
STP combined with anti-PD-1 antibody reduces chemokine expression and lactate secretion in tumors. **A** qRT-PCR analysis of mRNA levels in tumors treated with STP and anti-PD-1. **B** Lactate concentration in tumor tissues treated with STP and anti-PD-1. **C** Lactate concentration in conditioned medium of 4T1 cells treated with STP at indicated concentrations for 72 h. **D** CCK-8 assay detecting 4T1 cell proliferation inhibition by STP after 72 h

Given the reduction of myeloid cells across both blood and tumor compartments, we interrogated the relevant chemokine networks. We found that combination therapy significantly downregulated the expression of multiple chemokines in tumors (Fig. 3A). These downregulated chemokines, which included CCL4, CCL5, CCL8, CCL12, CXCL5, CXCL16, and CX3CL1, are implicated in the recruitment of both myeloid cells and T cells. Consistent with the established LDHA-inhibitory activity of STP, the combination treatment also reduced intratumoral lactate levels (Fig. 3B). In vitro studies confirmed that STP dose-dependently suppressed lactate production in 4T1 cells (Fig. 3C) and directly inhibited tumor cell proliferation, with a half-maximal inhibitory concentration (IC_50_) of 436 µM (Fig. 3D), suggesting a direct antitumor effect at relatively high concentrations.

### STP reprograms MDSC differentiation and attenuate MDSC immunosuppressive function

**Fig. 4:**
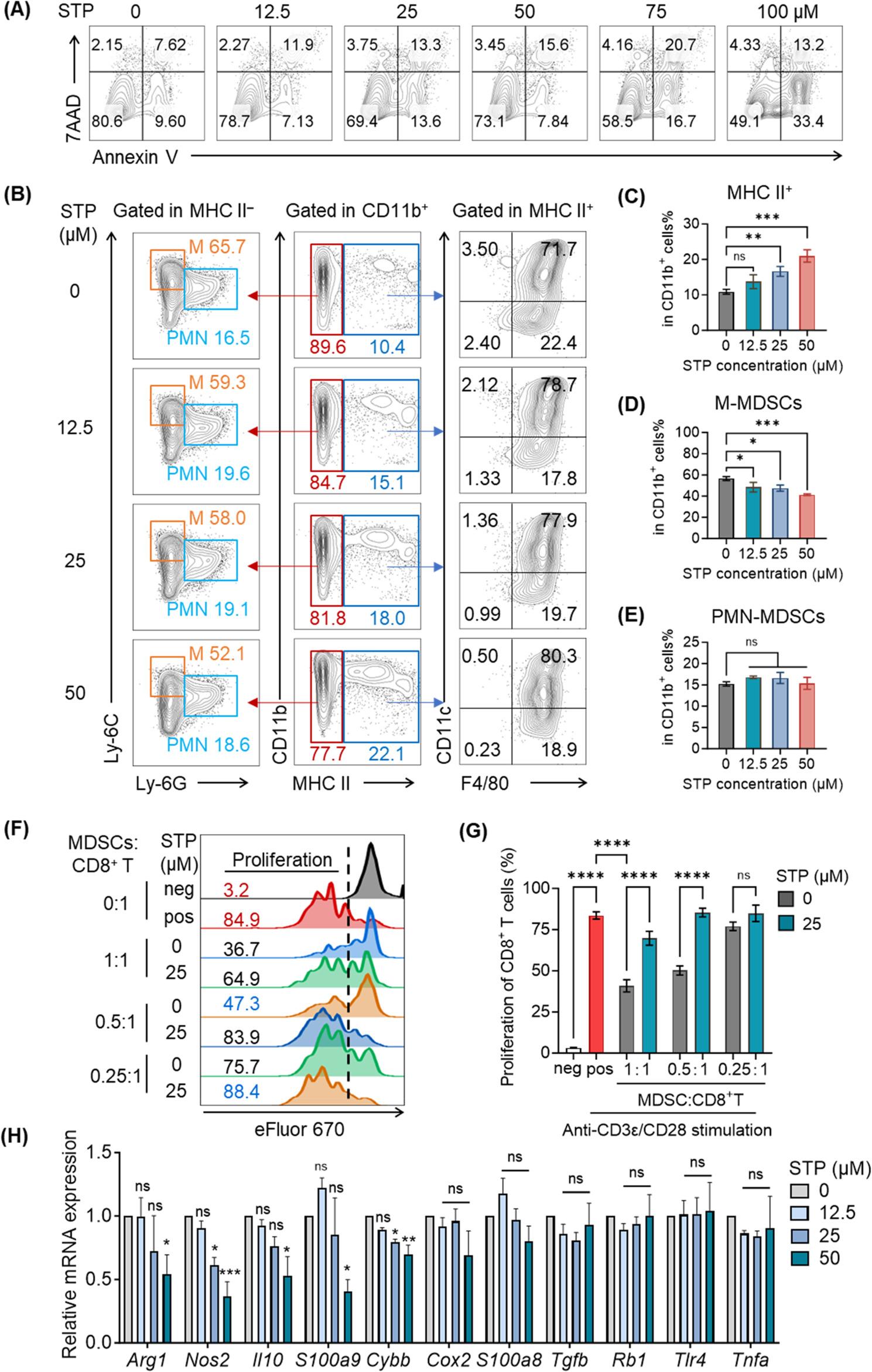
STP reprograms MDSC differentiation and attenuate MDSC immunosuppressive function. BM-MDSCs were generated by culturing bone marrow cells for 4 days with GM-CSF (40 ng/mL) and 20% (v/v) 4T1-conditioned medium in the presence of STP at indicated concentrations. **A** Apoptosis rates quantified by Annexin V/7-AAD staining in STP-treated BM-MDSCs. **B** Gating strategy to identify MHC II^+^ cells, M-MDSCs and PMN-MDSCs. **C**-**E** Proportion of MHC II^+^, M-MDSCs, and PMN-MDSCs. **F** Representative histograms of eFlour 670-labeled CD8^+^ T cell proliferation after co-culture with STP-treated BM-MDSCs at indicated ratios. **G** CD8^+^ T cell proliferation rates in co-culture assays with BM-MDSCs pre-treated with STP. Neg, negative control (T cell only, without anti-CD3ε/anti-CD28 antibodies stimulation); pos, positive control (T cell only, with anti-CD3ε/anti-CD28 antibodies stimulation). **H** qRT-PCR analysis of immunosuppressive genes in STP-treated BM-MDSCs

Given the observed reduction of MDSCs in STP-treated tumors, we further investigated whether STP directly regulates myeloid differentiation and function. Exposure to STP during GM-CSF/4T1-conditioned medium-driven MDSC differentiation induced concentration-dependent increased apoptosis (Fig. 4A). Flow cytometry revealed that STP dose-dependently elevated MHC-II expression concurrent with a reduction in M-MDSCs (Fig. 4B-E), suggesting promotion of terminal differentiation toward antigen-presenting cells. Functional assessment demonstrated that STP-treated MDSCs exhibited a significantly attenuated capacity to suppress CD8⁺ T-cell proliferation in co-culture assays across multiple ratios (Fig. 4F, G). Transcriptional profiling further confirmed this functional impairment, with STP dose-dependently downregulating key immunosuppressive genes (*Arg1*, *Nos2*, *Il10*, *S100a9*, *Cybb*; Fig. 4H). Collectively, these data demonstrate that STP directly reprograms MDSC differentiation toward immunostimulatory phenotypes while transcriptionally and functionally disabling their immunosuppressive machinery.

### STP reprograms M2 macrophage polarization

**Fig. 5:**
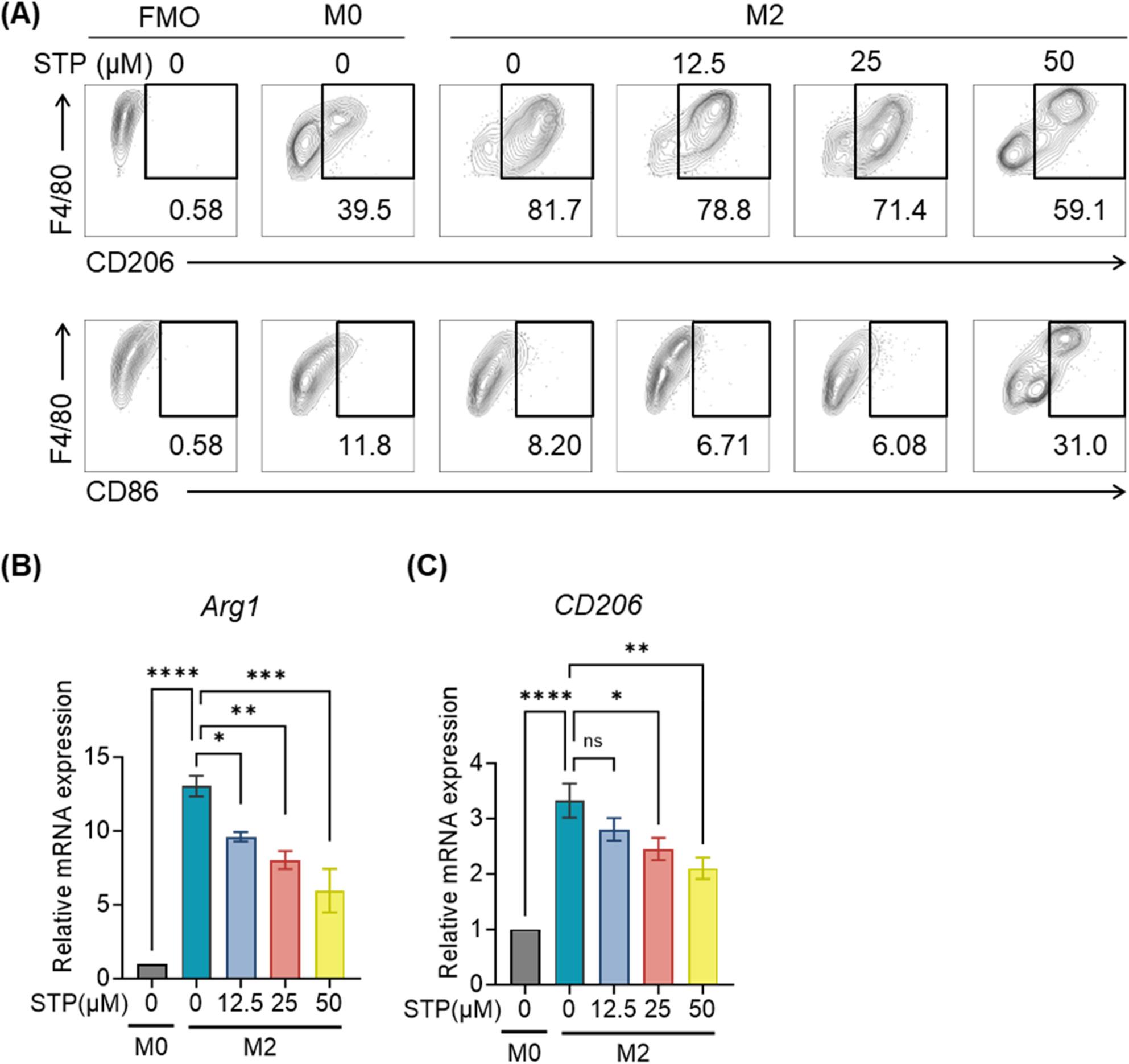
STP reprograms M2 macrophage polarization. BM-derived macrophages were generated by culturing bone marrow cells for 7 days with M-CSF (20 ng/mL) and 20% (v/v) 4T1-conditioned medium. Cells were polarized to M2 phenotype using IL-4 (100 ng/mL) and IL-13 (50 ng/mL) for 24 h, followed by STP treatment for 24 h. **A** Flow cytometry analysis of macrophage polarization status after STP treatment at indicated doses. M1 macrophages: F4/80^+^ CD86^+^; M2 macrophages: F4/80^+^ CD206^+^. **B**-**C** qRT-PCR analysis of Arg1 and CD206 mRNA levels in STP-treated M2-polarized BMDMs

Given the significantly reduced M2-like TAM frequencies following STP/anti-PD-1 combination therapy, we employed an in vitro BM-derived macrophage treated with STP during IL-4/IL-13-driven M2 polarization. STP dose-dependently skewed macrophages toward immunostimulatory phenotypes, showing increased CD86^+^ M1 populations and decreased CD206^+^ M2 subsets (Fig. 5A). Transcriptional profiling confirmed this phenotypic shift, showing significant downregulation of canonical M2 markers *Arg1* and *Cd206* (Fig. 5B, C). These data demonstrate that STP actively reprograms macrophage differentiation away from immunosuppressive M2 states toward antitumor M1 phenotypes, providing a mechanistic basis for its TME-remodeling activity.

### STP does not directly promote T cell activation

**Fig. 6:**
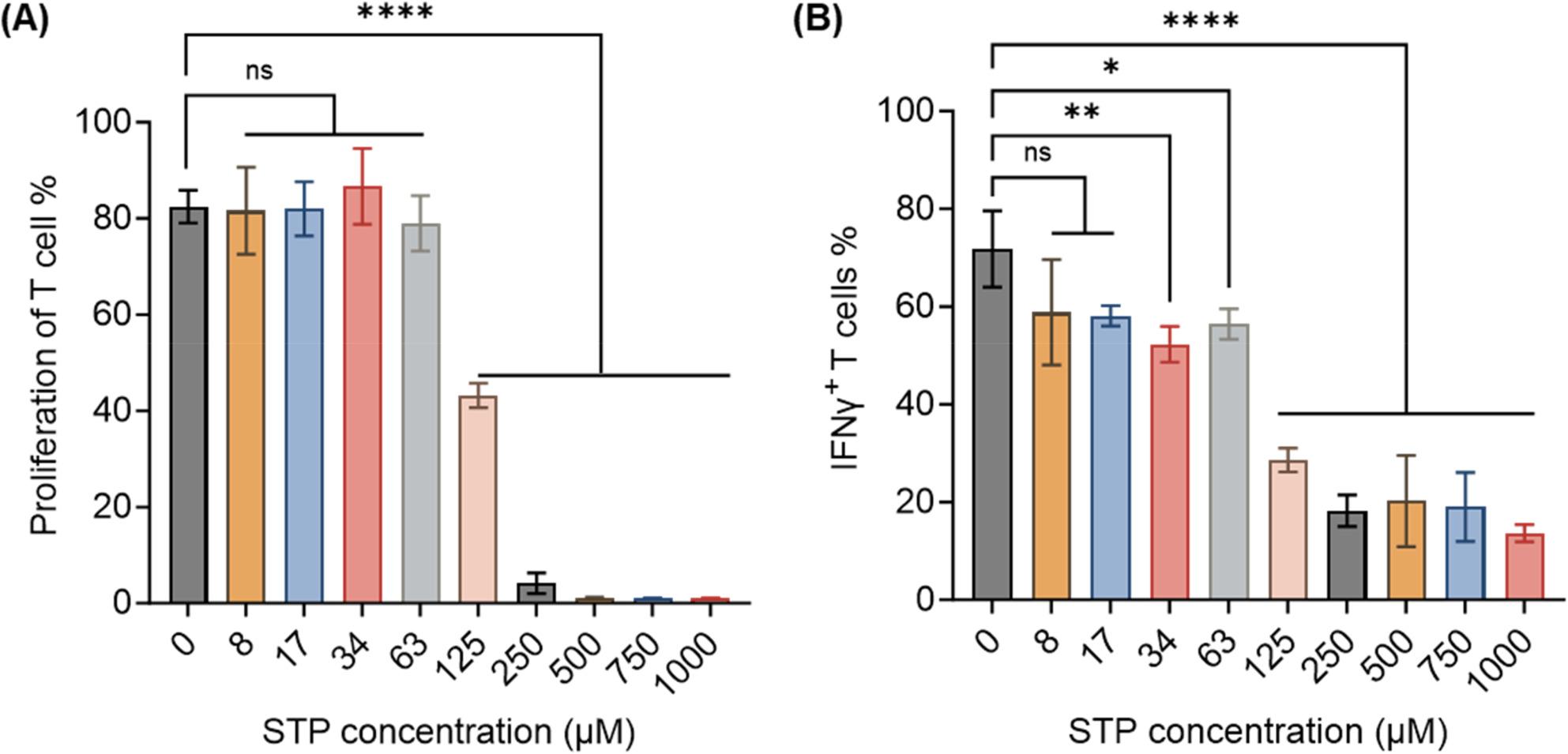
STP does not directly promote T cell activation. Magnetically sorted CD8^+^ T cells were stimulated with anti-CD3ε/CD28 and treated with STP at indicated concentrations for 72 h. **A** T cell proliferation measured by eFluor 670 dilution assay with indicated STP doses. **B** Flow cytometry analysis of IFN-γ expression in CD8^+^ T cells

To assess direct T cell modulation, isolated splenic CD8^+^ T cells were stimulated with CD3ε/CD28 antibodies in the presence of STP. No evidence of T cell activation or functional enhancement was observed at concentrations up to 63 µM. Paradoxically, at higher concentrations (≥ 125 µM), STP significantly suppressed T cell proliferation and IFN-γ production (Fig. 6A, B). These results definitively demonstrate that STP does not directly stimulate T cells and can even be inhibitory at pharmacologically relevant high doses. Therefore, the enhanced CD8^+^ T cell infiltration and activation observed in vivo likely result primarily from the alleviation of myeloid-mediated suppression and the reduction of intratumoral lactate.

## Discussion

**Fig. 7:**
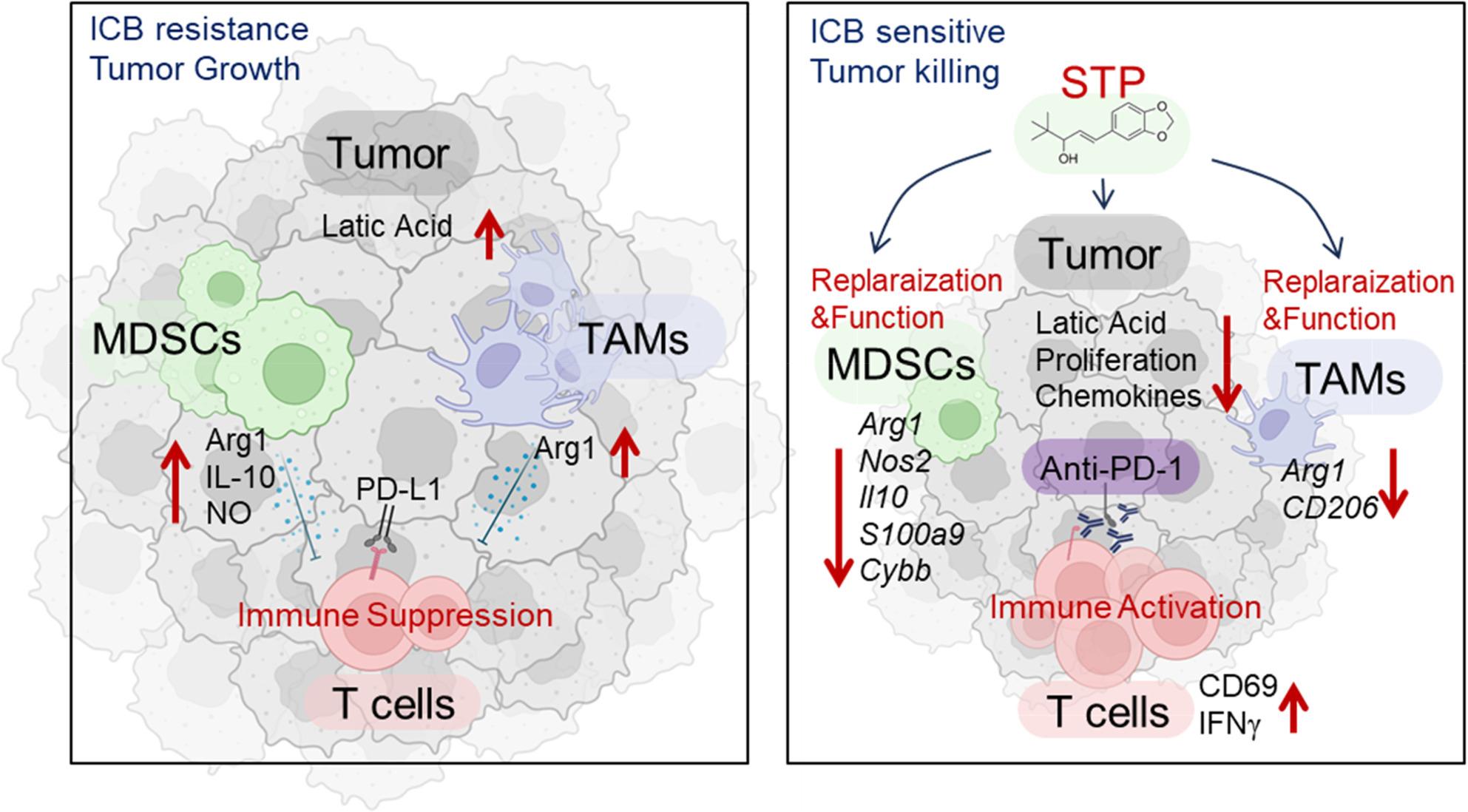
Schematic illustration of STP as a potent remodeler of the immunosuppressive tumor microenvironment that synergizes with anti-PD-1 therapy, providing a novel strategy to overcome ICB resistance

Our study demonstrates that STP synergizes with anti-PD-1 therapy to overcome ICB resistance, supporting its repurposing from an antiepileptic drug to an immunotherapy adjuvant. In the 4T1 model, combined STP and PD-1 blockade achieved significant tumor control by reprogramming the immunosuppressive TME, markedly attenuating tumor growth. STP simultaneously suppressed tumor proliferation and lactate secretion while directly reprogramming immunosuppressive myeloid cell. Specifically, STP reduced MDSC and M2-like TAM infiltration while promoting their differentiation toward immunostimulatory phenotypes (Fig. 7). This remodeling was associated with a broad downregulation of chemokines, including those implicated in T cell recruitment like CCL3-5 and CX3CL1, highlighting a complex reshaping of the TME. We propose that the efficacy of therapy stems not only from altering cell recruitment but primarily from converting the TME into a more permissive space, thereby liberating and potentiating the activity of tumor-infiltrating CD8⁺ T cells.

As a known LDHA inhibitor, STP significantly reduced lactate levels both in tumors and in 4T1 cell cultures. Lactate is increasingly recognized as a key metabolite that shapes immunosuppression [27]. It can drive PMN-MDSC differentiation via the Hes1/MCT2/c-Jun axis [28], enhance MDSC suppressive function through GPR81/mTOR/HIF-1α/STAT3 pathways [18], and promote M2 macrophage polarization via cAMP/PKA/CREB signaling [29]. While lactate predominantly fuels myeloid immunosuppression, evidence suggests it also supports stem-like properties in certain CD8⁺ T cell subsets important for sustained ICB response [30]. In our model, the potent synergy of STP with anti-PD-1 indicates that the benefits of disrupting the immunosuppressive network, thereby unleashing CD8⁺ T cell effector function, outweigh potential alterations to T cell metabolic states dependent on lactate.

Importantly, we provide novel evidence that STP directly attenuates myeloid immunosuppression independent from lactate modulation. The in vitro concentrations required for myeloid reprogramming were lower than those needed for direct tumor cytotoxicity, suggesting that immunomodulation is a primary and pharmacologically relevant mechanism. Furthermore, STP exhibited no intrinsic T cell-stimulatory capacity, indicating that the enhanced CD8⁺ T cell activity observed in vivo results primarily from the alleviation of immune suppression rather than direct T cell stimulation. The effects of STP appeared predominantly localized within the TME, the lactate-rich, hypoxic niche in where LDHA inhibition is most active. The more moderate systemic changes observed in peripheral blood likely represent secondary reflections of this intratumoral remodeling, rather than direct systemic immunomodulation. While our study focused on MDSCs and TAMs, the role of other myeloid populations, such as conventional dendritic cells in antigen presentation and T cell priming, warrants further investigation in the context of STP treatment.

## Conclusion

Our findings position STP as a clinically feasible strategy to remodel myeloid-rich, immunosuppressive TMEs, particularly in tumors with acquired PD-1 resistance. This effect is mediated by a dual-pathway mechanism: disrupting lactate-driven immunosuppression while directly attenuating myeloid cell function. Given its established safety profile and proven synergy, STP presents a compelling candidate for rapid clinical translation. Our work thus provides a strong rationale for repurposing STP as a myeloid-targeting adjuvant and supports its clinical evaluation in PD-1-resistant malignancies.

## Supplementary Information

Supplementary Material 1.

## Data Availability

The datasets used and/or analysed during the current study are available from the corresponding author on reasonable request.

## Abbreviations

BM: Bone marrow
ICB: Immune checkpoint blockade
LDHA: Lactate dehydrogenase A
MDSCs: Myeloid-derived suppressor cells
M-MDSCs: Monocytic MDSCs
NK: Natural killer
PMN-MDSCs: Polymorphonuclear MDSCs
STP: Stiripentol
TAMs: Tumor-associated macrophages
TME: Tumor microenvironment
Tregs: Regulatory T cells
