# Supplementary material for: Stiripentol directly attenuates immunosuppression of tumor-infiltrating myeloid cells to potentiate anti-PD-1 efficacy
Source: BMC Cancer. 2026 Feb 28;26:439. doi: 10.1186/s12885-026-15793-x (PMC13059454; doi:10.1186/s12885-026-15793-x)
Supplement: Supplementary file 1 — Supplementary Material 1. [file 12885_2026_15793_MOESM1_ESM.docx]

**Supplementary Table 1.** List of primer sequences used in qRT-PCR analysis.

| **Gene** | **Forward primer** | **Reverse primer** |
| --- | --- | --- |
| ***Gapdh*** | TGACCTCAACTACATGGTCTACA | CCGTGAGTGGAGTCATACTGG |
| ***Arg1*** | CCTATGTGTCATTTGGGTGGATG | GGTTGTCAGGGGAGTGTTGAT |
| ***Nos2*** | GGAGTGACGGCAAACATGACT | TCGATGCACAACTGGGTGAAC |
| ***Rb1*** | CAGGGCTGTGTTGACATCGGAGTA | TCCACGGGAAGGACAAATCTGTTC |
| ***S100a8*** | GGAAATCACCATGCCCTCTAC | GTCACTATTGATGTCCAATTCTCTGAA |
| ***S100a9*** | GCACAGTTGGCAACCTTTATG | TGATTGTCCTGGTTTGTGTCC |
| ***Il10*** | CTTACTGACTGGCATGAGGATCA | GCAGCTCTAGGAGCATGTGG |
| ***Cox2*** | TTCCAATCCATGTCAAAACCGT | CTGGTCAAATCCTGTGCTCAT |
| ***Cybb*** | CCTCTACCAAAACCATTCGGAG | CTGTCCACGTACAATTCGTTCA |
| ***Tlr4*** | AAATGCACTGAGCTTTAGTGGT | GGCTGTTTGTTCCCAAATCAGAA |
| ***Tgfb*** | CCGCAACAACGCCATCTATG | CCCGAATGTCTGACGTATTGAAG |
| ***Il6*** | CTGCAAGAGACTTCCATCCAG | AGTGGTATAGACAGGTCTGTTGG |
| ***Ccl2*** | TTAAAAACCTGGATCGGAACCAA | GCATTAGCTTCAGATTTACGGGT |
| ***Ccl3*** | TTCTCTGTACCATGACACTCTGC | CGTGGAATCTTCCGGCTGTAG |
| ***Ccl4*** | TTCCTGCTGTTTCTCTTACACCT | CTGTCTGCCTCTTTTGGTCAG |
| ***Ccl5*** | GCTGCTTTGCCTACCTCTCC | TCGAGTGACAAACACGACTGC |
| ***Ccl8*** | TCTACGCAGTGCTTCTTTGCC | AAGGGGGATCTTCAGCTTTAGTA |
| ***Ccl12*** | ATTTCCACACTTCTATGCCTCCT | GCATTAGCTTCAGATTTACGGGT |
| ***Cxcl1*** | CTGGGATTCACCTCAAGAACATC | CAGGGTCAAGGCAAGCCTC |
| ***Cxcl5*** | GTTCCATCTCGCCATTCATGC | GCGGCTATGACTGAGGAAGG |
| ***Cxcl16*** | CCTTGTCTCTTGCGTTCTTCC | TCCAAAGTACCCTGCGGTATC |
| ***Cx3cl1*** | ACGAAATGCGAAATCATGTGC | CTGTGTCGTCTCCAGGACAA |
| ***Tnfa*** | CCCTCACACTCAGATCATCTTCT | GCTACGACGTGGGCTACAG |

*Gapdh*: glyceraldehyde-3-phosphate dehydrogenase; *Arg1*: Arginase1; *Nos2*: Nitric oxide synthase2; *Rb1*: Retinoblastoma 1; *S100a8*: Recombinant S100 Calcium Binding Protein a8; *S100a9*: Recombinant S100 Calcium Binding Protein a9; *Il10*: Interleukin-10; *Cox2*: cyclooxygenase-2; *Cybb*: Cytochrome b-245 heavy chain gene; *Tlr4*: Toll-like Receptor 4; *Tgfb*: Transforming Growth Factor Beta*; Il6*: Interleukin 6*; Ccl2:* C-C Motif Chemokine Ligand 2*; Ccl3:* C-C Motif Chemokine Ligand 3*; Ccl4:* C-C Motif Chemokine Ligand 4*; Ccl5:* C-C Motif Chemokine Ligand 5*; Ccl8:* C-C Motif Chemokine Ligand 8*; Ccl12:* C-C Motif Chemokine Ligand 12*; Cxcl1:* C-X-C Motif Chemokine Ligand 1*; Cxcl5:* C-X-C Motif Chemokine Ligand 5; *Cxcl16:* C-X-C Motif Chemokine Ligand 16; *Cx3cl1:* C-X3-C Motif Chemokine Ligand 1*; Tnfa*: Tumor Necrosis Factor Alpha

**
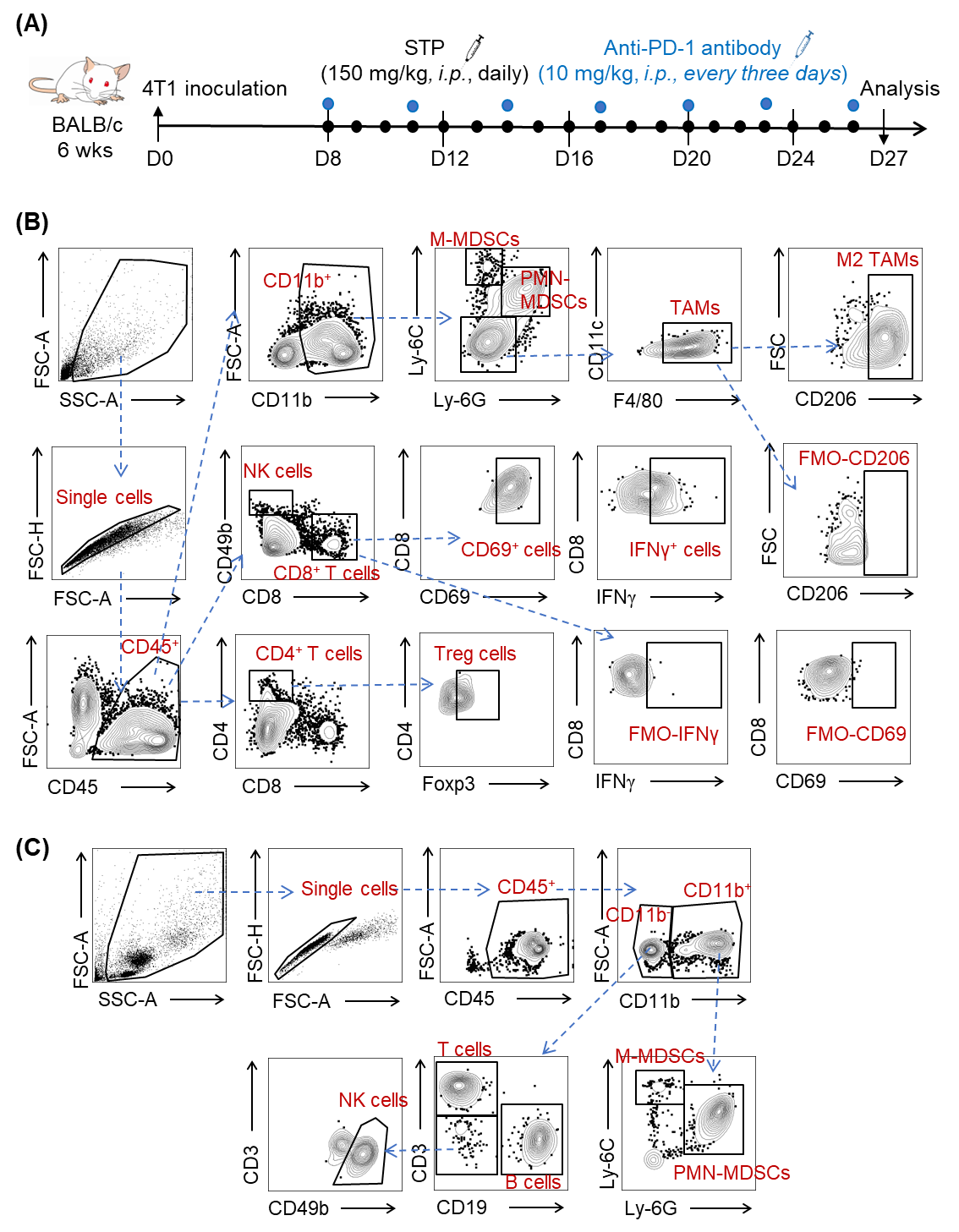
Supplementary Figure 1.** **(A)** Schematic representation of the experimental design: STP synergizes with anti-PD-1 treatment in an orthotopic 4T1 breast cancer model. **(B)** Gating strategy for tumor-infiltrating immune cell populations analyzed in Fig. 1. FMO controls were used as the reference for gating. **(C)** Gating strategy for peripheral blood immune cell populations analyzed in Fig. 2.
